# Neoadjuvant Therapy Effects on Invasive Mammary Carcinomas: Comparative Study of Pre- and Post-Therapy Cases and Assessment of Overall Survival Time

**DOI:** 10.1200/GO.22.50000

**Published:** 2022-05-05

**Authors:** Virginia Gomes, Carolina De Castro, Debora Balabram, Barbara Dos Santos, Cristiana Nunes

**Affiliations:** ^1^ Universidade Federal de Minas Gerais, Belo Horizonte, Brazil

## Abstract

To evaluate the pathological effects of neoadjuvant therapy (NT) on prognostic and predictive factors of invasive mammary carcinomas (IMC) and the impact of these changes on overall survival time.

**METHODS:**

Sixty-eight IMC diagnosed, treated with NT (2008-2016), with surgical excision at Hospital das Clínicas of UFMG showing pathological partial response. All samples were revised by an experienced breast pathologist, classified and graduated according to the WHO criteria. The histological tumor types were divided into three groups (no special type, lobular type and others). Estrogen and Progesterone receptors (ER and PR), HER2 and Ki67 status were evaluated through IHC. Statistical analysis was performed using the SPSS statistical program.

**RESULTS:**

Nineteen of 55 tumors (34.5%) showed changes in histological type (*P* = .024). Although not significant, 26/55 tumors had changes in histological grade, such as higher (27.3%) or lower grade (10.9%). Seven percent showed an increase in the percentage of inflammatory infiltrate (*P* = .004) and 35% had a decrease. Twenty-two of 55 (40%) of tumors showed ER changes and 20/55 (36,4%) showed PR changes. 96.4% of cases kept the same HER2 expression. 34.5% of tumors showed changes in the Ki67 expression. Ki67 distribution was different in post-therapy samples (non-normal distribution), diverting from expected (*P* = .014). There was an increase in Luminal A-like type (from 18.2% on pre-NT to 25.5% on post-NT samples) and Triple Negative type (from 34.5% on pre-NT to 45.5% on post-NT samples). These pathological changes found in post-neoadjuvant therapy samples did not affect the overall survival time of patients (median time of 44 months).

**CONCLUSION:**

Prognostic and predictive factors of IMC can change after neoadjuvant therapy. Reclassifying and retesting the residual samples is important to reevaluate treatment. There was no impact on overall survival of patients.

